# Broken Broaches in Pulp Canals

**Published:** 1887-02

**Authors:** 


### BROKEN BROACHES IN PULP CANALS.

A number of cases in which steel broaches had been broken off in the roots of teeth and left protruding through the foramen, to become the cause of serious disturbances, have lately come under our observation. In one, after an entire quiescence for a number of years, a pericemental inflammation of the most intense character supervened. The pain was extreme, and as after a week's suffering there were no symptoms of resolution by suppuration, the tooth was extracted. The cause was then revealed, in a piece of a broach which had been thrust quite through the end of the root and there left. The reason why there had been no septic symptoms was, that the steel had so perfectly closed the foramen that no septic organisms had found entrance, and the instrument was probably sterilized by antiseptics used at the time of the accident.

The root had been filled with cotton, skillfully packed, and the entire unfitness of this material for that purpose was demonstrated by its condition at the time of the extraction. It was completely disorganized and decayed. A portion of it was mounted in balsam for microscopical examination, and the fibres were found to be thoroughly broken up and quite rotted. A gold filling was over it, but had it not been for the sealing of the foramen of the tooth by the broken broach, the putrescent cotton must long before have brought about a periosteal abscess.

In another case, an abscess had existed for years, which finally resulted in necrotic tissue. The outer plate of the bone was opened and the dead tissue removed with a bur, but notwithstanding careful treatment, there was a refusal to return to a healthy condition. The tooth was extracted, when, through a widely divergent root of the bicuspid which had not been reached in the operation, a piece of broken bur was found protruding about three-sixteenths of an inch. Shall we be believed when we say that, after removal of the broach and the proper filling of the canals, this tooth was returned to its socket, and now, after some months, is in a serviceable condition? Yet such is the fact. Of course there was thorough drainage through the opening made by the operation, and it was quite possible to keep the territory aseptic when healing had commenced, by the proper remedies introduced into the wound, the tooth, in the meanwhile, being held rigidly in place by means of a retaining appliance.

Another case under observation was one in which a dentist had thrust a drill through an exceedingly short bicuspid tooth, and continued until he had entered the maxillary sinus, the result being an abscess of the antrum.

The lesson derived from these cases was, that it is necessary to exercise extreme caution lest a broach be thrust entirely through a tooth, not only carrying with it *debris* and septic matter, but running the risk of leaving a part of the instrument to be the cause of serious subsequent disturbances.

